# Expanding phenotype heterogeneity of *NARS2* by presenting subdural hematoma and parenchymal hemorrhage

**DOI:** 10.1002/jcla.24983

**Published:** 2023-11-10

**Authors:** Mehrnoosh Khodaeian, Fatemeh Bitarafan, Fatemeh Garrousi, Elham Amjadi Sardehie, Neda Pak, Sareh Hosseinpour, Marjan Shakiba, Masoumeh Falah, Masoud Garshasbi, Ali Reza Tavasoli

**Affiliations:** ^1^ Department of Medical Genetics DeNA Laboratory Tehran Iran; ^2^ Department of Medical Genetics Oslo University Hospital and University of Oslo 0450 Oslo Norway; ^3^ Pediatric Radiology Division, Children's Medical Center, Pediatrics Center of Excellence Tehran University of Medical Sciences Tehran Iran; ^4^ Myelin Disorders Clinic, Pediatric Neurology Division, Children's Medical Center, Pediatrics Center of Excellence Tehran University of Medical Sciences Tehran Iran; ^5^ Department of Pediatric Endocrinology and Metabolism, Mofid Children's Hospital Shahid Beheshti University of Medical Sciences Tehran Iran; ^6^ ENT and Head and Neck Research Center and Department, The Five Senses Health Institute, School of Medicine, Hazrat Rasoul Akram Hospital Iran University of Medical Sciences Tehran Iran; ^7^ Department of Medical Genetics, Faculty of Medical Sciences Tarbiat Modares University Teheran Iran; ^8^ Department of Neurology, Barrow Neurological Institute Phoenix Children's Hospital Phoenix Arizona USA

**Keywords:** asparaginyl‐tRNA synthetase 2, cerebral parenchymal hemorrhage, NARS2, subdural hematoma, whole exome sequencing

## Abstract

**Background:**

*NARS2* encodes mitochondrial Asparaginyl‐tRNA Synthetase 2, which catalyzes the aminoacylation of tRNA‐Asn in the mitochondria. To date, 24 variants have been reported in *NARS2* gene in 35 patients. The phenotypic variability of *NARS2‐*associated disorder is broad, ranging from neurodevelopmental disorders to hearing loss. In this study, we report some novel imaging findings in an Iranian patient suffering from epileptic encephalopathy, caused by a previously reported variant, c.500A > G; p.(His167Arg), in *NARS2*.

**Methods:**

The spectrum of clinical manifestations of two Iranian patients was investigated and genetic analysis was performed by Whole‐exome sequencing (WES). Additionally, we also reviewed the literature and summarized the phenotypes of previously reported patients with variants in the *NARS2* gene.

**Results:**

Here, we present the phenotypic and genetic features of 2 unrelated Iranian infants presented with neurodevelopmental delay, seizures, hearing impairment, feeding problems, elevated serum lactate levels in addition to subdural hematoma and cerebral parenchymal hemorrhage in the brain magnetic resonance imaging (MRI) of one of the patients. Genetic analysis revealed a biallelic missense variant in *NARS2*: c.500A > G; p.(His167Arg). We described the subdural hematoma and cerebral parenchymal hemorrhage of the brain for the first time.

**Conclusions:**

Our study provides new clinical findings, subdural hematoma, and parenchymal hemorrhage, in *NARS2*‐related disorders. Our findings along with previous studies provide more evidence of the clinical presentation of the disease caused by pathogenic variants in *NARS2*. Expanding the clinical spectrum increases the diagnostic rate of molecular testing and improves the quality of counseling for at‐risk couples.

## Graphical abstract

Summarized view of phenotypic and genetic findings of two patients carrying c.500A > G variant in *NARS2*.

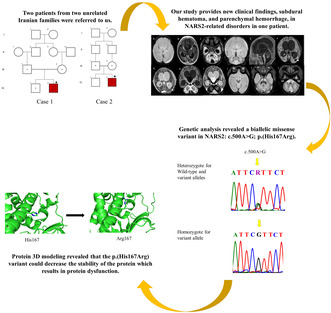

## 1 INTRODUCTION

More than 250 variants in both nuclear and mitochondrial genes have been reported in a heterogeneous group of disorders presented with a wide range of clinical manifestations, called mitochondrial disorders.^1^ Among the nuclear genes, those coding for the mitochondrial aminoacyl‐tRNA synthetases (mt‐aaRSs) have been shown to be commonly related to human mitochondrial disorders. These enzymes charge mitochondrial tRNA molecules with their cognate amino acids. Although most mitochondrial proteins are encoded by the nuclear genome in the cytosol, a mitochondrial translation system is necessary to translate the 13 mitochondrial polypeptide subunits that are vital for oxidative phosphorylation.^2^, ^3^ The linkage of amino acids to their cognate tRNAs, which is mediated by mt‐aaRSs is a crucial step in protein synthesis in mitochondria.^4^, ^5^ Deficiency of asparaginyl‐tRNA synthetase (NARS2), NP_078954.4, as a member of mt‐aaRSs group has been reported in association with human disorders including both autosomal recessive nonsyndromic hearing loss type 94 (MIM: 618434) and combined oxidative phosphorylation deficiency 24 (COXPD24; MIM: 616239) that affects multiple organs mostly the central nervous system.^6^, ^7^, ^8^

Up till now, 24 variants in *NARS2* gene (NM_024678.6) in 35 cases including 20 patients carrying homozygous and 15 patients carrying compound heterozygous variants have been reported in the literature and presented with a wide spectrum of clinical symptoms even among affected individuals carrying the same variants. All of the data related to families and variants are summarized in Table 1.

**TABLE 1:** Summarized review of the phenotypic and genetic findings of all identified patients with variants in *NARS2.*

NARS2 variants		Phenotype	NARS2 domain	Pathogenicity	Important phenotype			Paraclinical results			Other features	Survival	Ref.
Nucleotide	Protein			Franklin ACMG classification	Seizure	Intellectual impairment	Muscle disorders	MRI	EEG	Lab. Tests			
c.500A > G (homozygote)	p.(His167Arg)	COXPD24 (OMM: 616239)	Catalytic	VUS	Yes	Yes	Yes	Cerebral atrophy	Burst suppression pattern	Increased serum lactate	Hearing impairment, microcephally, cerebral atrophy	Alive (4 y)	9
c.500A > G (1st case) (homozygote)	p.(His167Arg)	COXPD24	Catalytic	VUS	Yes	Yes	Yes	Subdural hematoma, cerebral parenchymal hemorrhage	Generalized epileptic discharge, posterior polymorphic delta activity, and diffuse fast activity	Elevated lactate in blood	Panhypopituitarism, hepatomegaly, Hypothyroidism, hypoparathyroidism, speech problems, feeding problems, horizontal nystagmus, Hearing impairment	Dead (2 y)	This Study
c.500A > G (2nd case) (homozygote)	p.(His167Arg)	COXPD24	Catalytic	VUS	Yes	Yes	Yes	Normal	Not available	Increased serum lactate	Feeding problems, hearing impairment	Dead (2 y)	This Study
c.641C > T (homozygote)	p.(Pro214Leu)	Alpers	Catalytic	Likely Pathogenic	Yes	Yes	Yes	Cerebral atrophy	Abnormal	Increased lactate	Hearing impairment, optic atrophy, microcephaly, urinary retention	Dead (6 y)	10
c.641C > T (homozygote)	p.(Pro214Leu)	Alpers	Catalytic	Likely Pathogenic	Yes	Yes	Yes	Cerebral atrophy	Abnormal	Increased lactate	Hearing impairment, optic atrophy, microcephaly, urinary retention	Dead (16 y)	10
c.641C > T (homozygote)	p.(Pro214Leu)	Alpers/Leigh syndrome	Catalytic	Likely Pathogenic	Yes	Yes	Yes	Abnormal signal in basal ganglia, cortical atrophy	Abnormal	Increased lactate	Renal dysfunction	Alive (25 y)	6
c.822G > C (homozygote)	p.(Gln274His)	COXPD24	Catalytic	VUS	No	No	Yes	Not available	Not available	Not available	Mitochondrial myopathy	Alive (34 y)	11
c.822G > C (homozygote)	p.(Gln274His)	COXPD24	Catalytic	VUS	Yes	Yes	Yes	Not available	Not available	Not available	Epilepsy	Alive (26 y)	11
c.822G > C (homozygote)	p.(Gln274His)	COXPD24	Catalytic	VUS	No	Yes	Yes	Not available	Not available	Not available	Urinary incontinence, kidney disorder, scoliosis	Alive (29 y)	12
c.969 T > A c.1142A > G (compound heterozygote)	p.(Tyr323*) p.(Asn381Ser)	Leigh syndrome	Catalytic Catalytic	Pathogenic Pathogenic	Yes	No	Yes	Hyperintensities	Not available	Increased lactate levels	Hearing loss, laryngomalacia, pharyngeal hypotonia	Dead (15 m)	13
c.969 T > A c.1142A > G (compound heterozygote)	p.(Tyr323*) p.(Asn381Ser)	Leigh syndrome	Catalytic Catalytic	Pathogenic Pathogenic	Yes	No	Yes	Restricted diffusion left basal ganglia	Not available	Not available	Hearing loss, feeding difficulties	Dead (6 m)	13
c.637G > T (4 patients) (homozygote)	p.(Val213Phe)	Autosomal recessive deafness‐94	Catalytic	Likely pathogenic	Not available	Not available	Not available	Cerebral atrophy, cerebellar lesion, basal ganglia lesion	Abnormal	Not available	Nonsyndromic hearing loss, Premature menopause	Alive (26‐40y)	13
c.641C > T ex. 8–9 del (compound heterozygote)	p.(Pro214Leu) ‐	Mitochondrial disorder	Catalytic Catalytic	Likely Pathogenic	Not available	Not available	Not available	Abnormal	Not available	Not available	Intrauterine growth restriction, perinatal insult, gastrointestinal reflex, anemia, lactic acidosis, ketosis, hyperglycemia	Not available	14
c.707 T > G c.594 + 1G > A (compound heterozygote)	p.(Phe236Cys) p.(Asp172_Glu198)	COXPD24	Catalytic Catalytic	VUS Likely Pathogenic	Yes	Yes	Yes	Diffuse atrophy	Abnormal	High serum lactate	Hearing impairment, microcephaly, flaccid quadriplegia	Alive (8 y)	9
c.707 T > G c.594 + 1G > A (compound heterozygote)	p.(Phe236Cys) p.(Asp172_Glu198)	Alpers	Catalytic Catalytic	VUS Likely Pathogenic	Yes	Yes	Yes	Cerebral atrophy	Abnormal	High serum lactate	Hearing impairment, microcephaly, flaccid quadriplegia, optic atrophy, renal dysfunction	Alive (1 y)	9
c.151C > T c.1184 T > G (compound heterozygote)	p.(Arg51Cys) p.(Leu395Arg)	COXPD24	Anti codon binding Catalytic	VUS Likely Pathogenic	Yes	Yes	Yes	Diffuse atrophy	Abnormal	High serum lactate	Hearing loss, hemiparesis, flaccid quadriplegia, pharyngeal hypotonia	Alive (2 y)	9
c.167A > G c.631 T > A (compound heterozygote)	p.(Gln56Arg) p.(Phe211Ile)	Alpers	Anti codon binding Catalytic	VUS Likely Pathogenic	Yes	No	No	Cerebral atrophy, subdural effusions, cerebral restricted diffusion	Abnormal	Increased serum lactate	Auditory neuropathy	Dead (5.5 m)	15
c.167A > G c.631 T > A (compound heterozygote)	p.(Gln56Arg) p.(Phe211Ile)	Alpers	Anti codon binding Catalytic	VUS Likely Pathogenic	Yes	No	No	Cerebral atrophy, subdural effusions, cerebral restricted diffusion	Abnormal	Not available	Auditory neuropathy	Dead (9 m)	15
c.545 T > A (homozygote)	p.(Ile182Lys)	COXPD24	Catalytic	Likely Pathogenic	Yes	Yes	Yes	Not available	Bilateral synchronous spike, polyspike waves	Not available	Auditory neuropathy, progressive ataxia, clubbed fingers, brachymetatarsia	Alive (17 y)	16
c.545 T > A (homozygote)	p.(Ile182Lys)	COXPD24	Catalytic	Likely Pathogenic	Yes	Yes	Yes	Not available	Bilateral synchronous spike, polyspike waves	Not available	Auditory neuropathy	Alive (28 m)	16
c.1253G > A c.1300C > T (compound heterozygote)	p.(Arg418His) p.(Leu434Phe)	COXPD24	Catalytic Catalytic	VUS VUS	Yes	Not available	Not available	Not available	Not available	Not available	Not available	Not available	17
c.731C > G c.1351C > T (4 patients) (compound heterozygote)	p.(Ala244Gly) p.(Arg451Cys)	Leigh syndrome	Catalytic Catalytic	VUS Likely Pathogenic	Not available	Not available	Not available	Not available	Not available	Not available	Not available	Not available	18
c.951C > T (homozygote)	p.(Asn317Asn)	Reversible COX deficiency	Catalytic	VUS	No	Yes	Yes	Asymmetry of the hippocampus	Abnormal	High serum lactic acid	Hearing loss, hypotonia	Alive (22 y)	19
c.658A > G (6 patients) (homozygote)	p.(Met220Val)	HL Or NSHL	Catalytic	Likely Pathogenic	Not available	Not available	Not available	Not available	Not available	Not available	Hearing loss, non‐syndromic hearing loss	Not available	8
c.83_84del c.1339A > G (compound heterozygote)	p.(Leu28Glnfs*17) p.(Met447Val)	Fatal refractory status epilepticus	Catalytic	Likely Pathogenic Likely Pathogenic	Yes	Not available	Yes	Progressive cortical and periventricular brain atrophy	Focal discharges, multifocal spikes and sharp waves	Not available	Bradycardia, renal failure	Dead (14 m)	7
c.1141A > G c.1290G > C (compound heterozygote)	p.(Asn381Asp) p.(Trp430Cys)	COXPD24	Catalytic Catalytic	Likely Pathogenic VUS	Yes	Yes	Yes	Normal	Slow waves, irregular spikes in the central, parietal, and temporal regions	Increased alanine transaminase, aspartate transaminase, alkaline phosphatase, lactate dehydrogenase	Hearing impairment	Dead (6 m)	20

Most of the patients who carried pathogenic variants in *NARS2* presented with an increased level of serum lactate, refractory epileptic seizures, sensorineural hearing impairment, intellectual disability, and global psychomotor delay or regression. Other features include progressive microcephaly, renal dysfunction, optic atrophy, cortical visual impairment, spastic quadriplegia, hemiparesis, flaccid quadriplegia, urinary retention, amyotrophy, hyperCKemia, excessive fatigue, laryngomalacia, pharyngeal hypotonia, feeding difficulties, ataxia, brachymetatarsia, hallux valgus, hypotonia, upper limb tremor, clubbed fingers, mitral valve prolapse developmental delay, and brisk deep tendon reflexes, and premature menopause in females was observed in some cases.^6^, ^16^ The most commonly reported initial phenotype based on age of disease onset is summarized in Table 2.

**TABLE 2:** The most common reported clinical and symptoms in NARS2‐associated disorders.

Age of Onset	Number of cases	Most common presentations	Other reported findings (Rare)
Perinatal/Neonatal (Initial phenotype)	3	Alpers phenotype, opisthotonus posturing, poor head control, feeding problem	Spastic quadriplegia
Infantile (Initial phenotype)	13	Alpers/Leigh phenotype, hearing and visual impairment, psychomotor delay, seizure, microcephaly, non‐syndromic hearing loss, absent DTRs*	Urinary retention, feeding problem, limb spasticity, nystagmus, hallux valgus, clubbed fingers, mitral valve prolapse Flaccid or spastic quadriplegia
Childhood (Initial phenotype)	2	Myopathy, ptosis, fatigue, epilepsy, Intellectual disability	Dysarthria, Amyotrophy HyperCKemia

*Note*: *DTRs: Deep Tendon Reflexes.

In this study, we investigated two unrelated Iranian male infants from two individual families who presented with epileptic encephalopathy. Genetic findings revealed a reported biallelic *NARS2* variant in both cases; c.500A > G (p.His167Arg). Brain magnetic resonance imaging (MRI) of one patient showed novel imaging findings including subdural hematoma, cerebral parenchymal hemorrhage, and cerebellar cortical restriction in diffusion weighted sequences (DWI) that were not reported previously.^9^

## 2 MATERIALS AND METHODS

### 2.1 Ethical statements

Written informed consent was obtained from all participants or their respective guardians. The study was approved by the ethical committee of Tehran University of Medical Sciences as well as the ethical committee of the National Institute for Medical Research Development (NIMAD) of Iran under the codes of ID: IR.NIMAD.REC.1397.508 and ID: IR.NIMAD.REC.1399.066, respectively.

### 2.2 Case presentation

**FIGURE 1:**
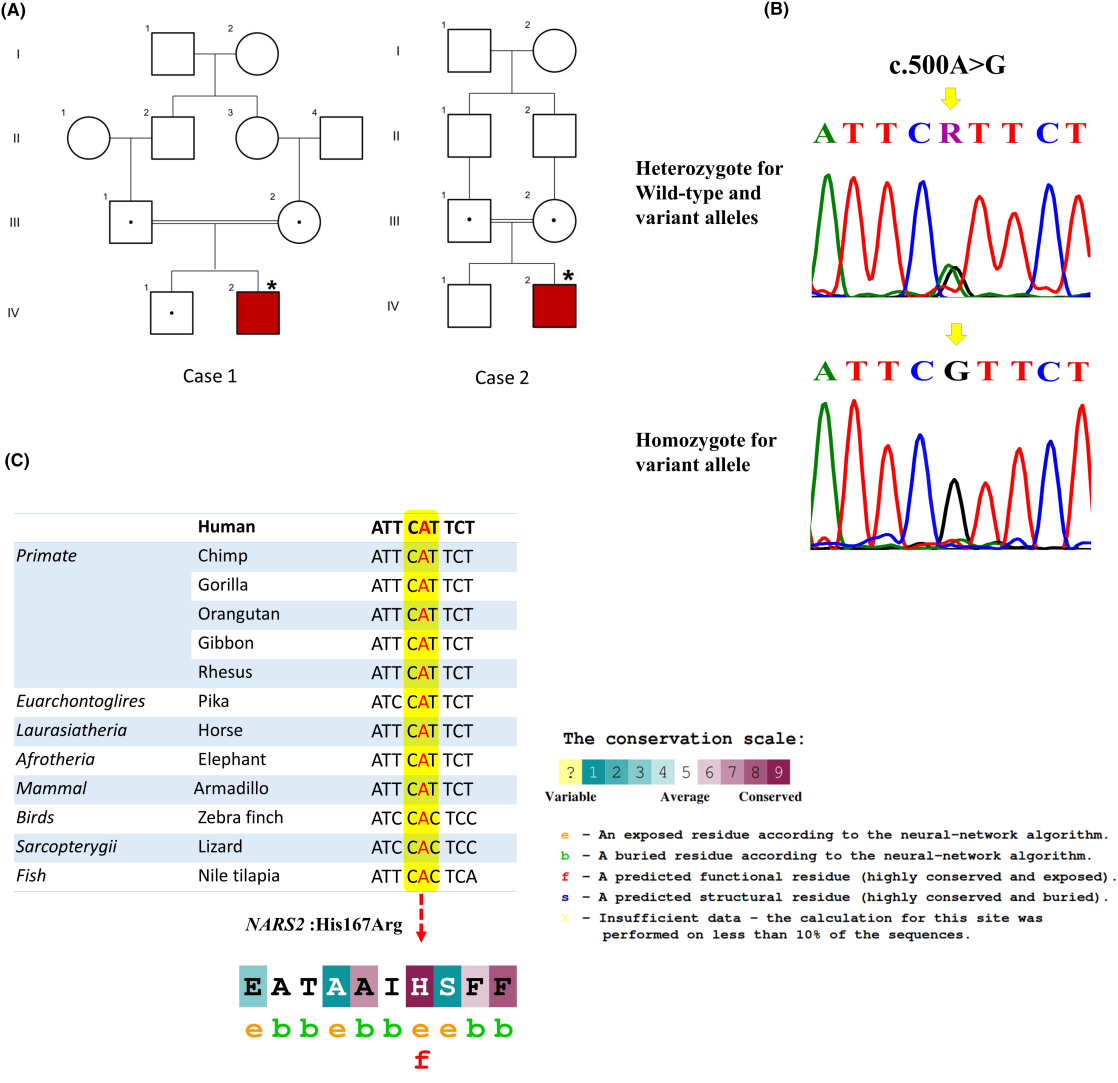
The Pedigree of the investigated families, Sanger sequencing chromatograms, and multiple sequence alignment. (A) The Pedigree of the investigated families and in these pedigrees, an asterisk (*) indicates the patient that was selected for performing WES. Red symbol: affected and homozygous for the variant; dotted symbols: carriers for the variant; squares: males; circles: females; parallel lines: consanguineous marriage. (B) Sanger sequencing chromatograms showing nucleotide sequences of *NARS2* in the regions of c.500A > G which is found in the families. (C) UCSC database used to show the multiple sequence alignment displaying evolutionary conservation of c.500A nucleotide and His167 (H) in the *NARS2* gene among different species. Meanwhile, the amino acid sequence of NARS2 protein colored according to the conservation scores provided by the ConSurf server.

Here, we report two patients from two unrelated Iranian families who presented with epileptic encephalopathy and were referred to Myelin Disorders Clinic, Children's Medical Center, Tehran, Iran, and DeNA Laboratory, Department of Medical Genetics, Tehran, Iran (Figure 1A). The written informed consent was obtained from all participants or their legal guardians. Participants also provided written informed consent for publication of their information included in this paper.

### 2.3 Clinical outcome

#### 2.3.1 Patient 1

The patient was a 2‐year‐old boy, with suspected genetic epileptic encephalopathy, who was first visited at pediatric intensive care unit (PICU) due to frequent seizures, poor general condition, and decreased level of consciousness. He was the second child of consanguine parents and was born at term pregnancy through an uneventful normal vaginal delivery without any significant event during prenatal periods. His birth weight and head circumference (HC) was 3100 g (*z* score = 0) and 35.5 cm (*z* score = 1), respectively. He had a healthy five‐year‐old brother.

His parents first noted a blunted response to environmental stimuli, such as acoustic stimulus, and social events, such as lack of social smile, at 2 months of age. However, he was able to hold his neck. Then, he developed non‐fixed transient episodes of unilateral torticollis and focal motor and tonic spasm seizures at 3.5 months of age. He was hospitalized and his initial laboratory workups indicated only a low total serum calcium level (total calcium level of 6 mg/dL, normal range: 7.8–10 mg/dL), phosphorus level of 6.8 mg/dL (normal range: 4.5–6.5 mg/dL), magnesium level of 2 mg/dL (normal range: 1.5–5.5 mg/dL), alkaline phosphatase of 628 (normal range: 140–720 IU/L), serum lactate level of 18.7 mmol/L (normal range; 4.5–19.8 mmol/L), and normal level of serum albumin and ammonia. He was prescribed calcium supplements in addition to phenobarbital, which resulted in seizure control lasting for a few weeks just based on clinical observation. However, Electroencephalogram (EEG) was not performed due to family dissatisfaction. On examination at discharge, motor and cognition delays were detected.

**FIGURE 2:**
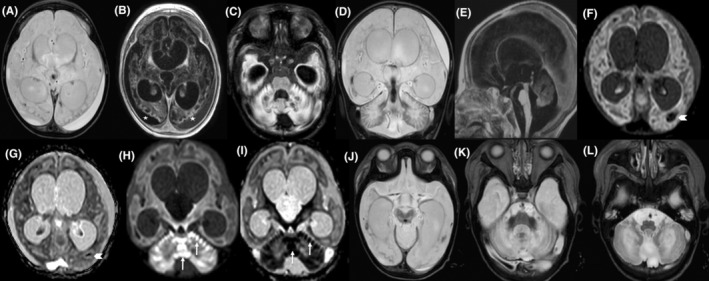
Brain MRI of a 16‐month old boy (case 1) with mutation in *NARS2*. Axial T2‐Weighted, T1‐Weighted and FLAIR sequences, respectively (A–C), and coronal T2‐Weighted image (D) show significant supra and infratentorial parenchymal destruction as white matter cystic change and vacuolization with involvement of U fibers, involvement of cerebral and cerebellar cortex, abnormal T2 hyperintensity and T1 hypointensity, cystic change and atrophy of basal ganglia and thalami, involvement of dentate nuclei and ventriculomegaly. The cerebral atrophy and secondary subdural hematoma in different ages are shown by white stars‐B. In sagittal T1‐Weighted image (E) note the cystic change and abnormal signal intensity of vermis. Axial diffusion weighted images (DWI) images (F, H) and ADC map sequence (G, I) demonstrate parenchymal hemorrhage (white arrowheads) as blooming artifact and restriction of vermis and cerebellar cortex (white arrows) representing acute phase in restricted areas. Axial T2‐Weighted views (J–L) show mid brain, pons and medulla oblongata involvement, respectively.

After a few weeks, the patient's seizures flared up with severe restlessness attacks and crying followed by apnea that was lasting up to 1 min following a mild upper respiratory tract infection and fever. These events resulted in a second hospitalization. His seizures evolved to mixed‐type ones consisting of focal motor clonic seizures with lateral gaze, head version, and focal to bilateral and multifocal motor type seizures that ultimately led to PICU admission at the age of 9 months. The EEG depicted generalized epileptic discharge, posterior polymorphic delta activity, and diffuse fast activity due to the benzodiazepine effect (Figure 2). During hospitalization, he encountered a poor general condition and infrequent repetition of seizures. In addition, he experienced severe electrolyte imbalances, including low serum total calcium of 6.5 mg/dL, low magnesium level of 1 mg/dL, hyponatremia (Na:106, range: 135–145 mEq/L), mild hypokalemia (3 mmol/L, range: 3.6–5.2 mmol/L), low serum level of 25–OH– Vit D3, (<8 nmol/L with a normal range of above 30 nmol/L), and low serum ACTH level of 3 pg/mL (normal range of 7.1–56.3 pg/mL). Endocrinology consultation suggested panhypopituitarism. Therefore, a low dose of hydrocortisone tablet was started which was helpful to maintain serum electrolytes. Other laboratory tests indicated severe microcytic anemia with hemoglobin level of 5 mg/dL (range: 9.5–13 g/dL) and mean corpuscular volume (MCV) of 71 fl (range: 75–108), thrombocytopenia (13 × 10^3^, normal range of 150–450 × 10^3^), hypoparathyroidism with parathyroid hormone (PTH) level of 607 pg/mL (range: 15–65 pg/mL), and also central hypothyroidism (TSH: 10.7, range: 1.7–7.5 μM). In addition, serum lactate levels were recorded between 11 and 29 mmol/L, and cerebrospinal fluid (CSF) lactate level was 16 mmol/L (normal range; less than 12 mmol/L). He never had hyperkalemia. In addition, the patient had experienced episodes of sinus bradycardia with a heart rate of 30–35 beats per minute, occasionally for which cardiological investigations including echocardiography and Holter monitoring were done with normal results. Furthermore, the patient encountered persistent high blood pressure up to a maximum level of 200 mmHg and diastolic blood pressure of 110 mmHg. On examination, hepatomegaly with a liver span of 3 cm below the rib edge was also detected. His admission at PICU and then general pediatric neurology ward lasted for 7 months, and a variety of anti‐seizure medications including phenobarbital, levetiracetam, lamotrigin, phenytoin, Sabril, clonazepam was prescribed to control his seizures. All electrolyte abnormalities were corrected with a supplementary drug and a low dose of hydrocortisone. Hypothyroidism, hypocalcemia, and hypoparathyroidism were controlled with oral levothyroxine, calcium, and vitamin D supplements. Hypertension was controlled with amilodipine. Occupational and speech therapy was started to improve his swallowing and gross motor abilities. The patient's seizure frequency was reduced to <50% and finally, after a longstanding hospitalization, he was discharged at 16 months of age.

Regarding his developmental milestones, the patient had never achieved normal motor, speech and cognitive milestones. In addition, he had experienced motor and cognition regression and loosing of fix and follow ability and social smile during the long‐standing hospitalization. He gradually lost the ability to swallow as he had constant drooling. Therefore, feeding was established through a nasogastric (NG) tube. Due to persistent abdominal distension, a full set of gastroenterological investigations were also performed, which confirmed gastroenterological autonomic dysfunction and it was treated with Motilium syrup. Besides that, abnormal movement, especially generalized four limbs dystonia, developed after 1 year of age, which was treated with trihexyphenidyl and baclofen, which resulted in significant improvement.

The first brain MRI at 16 months old revealed severe subcortical and deep cerebral, cerebellar and peridentate white matter involvement, symmetric bilateral basal ganglia involvement, severe brain atrophy with large cystic necrotic areas in cerebral hemispheres and severe thinning of the corpus callosum. Furthermore, subdural hematoma, cerebral parenchymal hemorrhage, and cerebellar cortical restriction in diffusion‐weighted sequences of brain MRI were detected that were novel imaging findings in this disorder (Figure 2).

On his last physical examination at 2 years old, he had a weight of 8 kg (below the 3rd percentile, *z* score: −3) and an HC of 44 cm (below the 3rd percentile, *z* score: −3). He had poor cognition abilities with no purposeful fix and follow, low‐beat horizontal nystagmus, and normal pupils' reaction to light. The gag reflex was not satisfactory. He had obvious drooling and feeding was with an NG tube. Due to prolonged feeding with NG tube, Percutaneous Endoscopic Gastrostomy (PEG) was placed for him. Examination of other cranial nerves was not feasible. He was experiencing occasional dystonic postures. Deep tendon reflexes were exaggerated (4+) with clonus and plantar reflexes were bilaterally upward. Moreover, he had moderate four limbs spasticity, especially in lower limbs, and notable hand fisting. No scoliosis, joint deformity, or contracture was detected. His gross motor ability using Gross Motor Function Classification System (GMFCS) score was calculated as five out of five (5/5). On general examination, the skin was normal without any congenital or acquired rash. Mild hepatomegaly was also detected. He had normal heart and respiratory rates and blood pressure.

Other basic metabolic tests, including serum creatine phosphokinase (CPK) and aldolase, urine organic acids and acylcarnitine profiles, and metabolic screen test (MS/MS), were all normal. Ophthalmologic consultation at age of 18 months was normal. An auditory brain stem response test (ABR) showed moderate central sensory motor hearing loss in both ears. Renal ultrasonography was normal. A repeated EEG showed a low voltage background and was not remarkable for epileptiform discharge (not shown). Finally, he died at the age of 2 years due to respiratory system failure.

#### 2.3.2 Patient 2

The patient was a male patient who was 6 months old at first visit and born in a family with first cousin parents without any complications during pregnancy. He revealed seizures at 3 months of age for the first time and motor delay. He suffered from bilateral sensorineural hearing loss, neurodevelopmental delay, truncal hypotonia, and epileptic encephalopathy. Hypocalcemia, hypomagnesemia, and increased serum lactate up to 30 mmol/L were also observed. He also presented with swallowing and feeding problems resulting in using PEG for feeding. His first brain MRI was normal at 6 months of age, and finally, he died as a result of respiratory system failure, at the age of 1 year. No additional clinical information, including follow‐up notes after the first visit at 6 months of age, was available at the time of study.

### 2.4 Genetic findings

Peripheral blood samples were obtained from the affected patients and their parents. A High Pure PCR template preparation kit (Roche; Product No 11814770001) was used to extract genomic DNA. To enrich approximately 60 Mb of the Human Exome from fragmented genomic DNA, Agilent's SureSelect Human All Exon V6 kit was used. The generated library was sequenced on an Illumina Hiseq 4000 platform to obtain an average coverage depth of 100. Typically, 97% of the targeted bases were covered >10. An end to end in‐house bioinformatics pipeline including base calling, alignment of reads to GRCh37/hg19 genome assembly, primary filtering out of low‐quality reads and probable artifacts, and subsequent annotation of variants was applied. Evaluation was focused on coding exons along with flanking ±20 intronic bases. All disease‐causing variants reported in HGMD and ClinVar as well as all variants with minor allele frequency (MAF) of less than 1% in publicly available mutation and polymorphism databases such as 1000 genome project, ExAC (Exome Aggregation Consortium), ESP (Exon Sequencing Projects), gnomAD, and Iranome database were considered.

After several filtering steps, whole‐exome sequencing (WES) analysis was done based on previous works^16^ and revealed a previously reported pathogenic homozygote missense variant in the *NARS2* gene, c.500A > G (p.His167Arg), compatible with the diagnosis of autosomal recessive COXPD24 (OMIM: 616239) disorder in both patients. In order to check the segregation of the variant in the family, sanger sequencing on both patients and their parents was performed (primers and conditions are available upon request). In both families, the parents were identified as heterozygous for the variant (Figure 1B). Using ConSurf (http://www.consurf.tau.ac.il)^21^ and UCSC database,^22^ the evolutionary conservation of the detected variant was analyzed, and it was shown that the variant is highly conserved and exposed (Figure 1C).

As the c.500A > G variant was previously reported, in different databases such as Franklin ACMG automated Classification, it is classified as a Variant with Unknown Significance (VUS), but according to the recommendation of the ACMG for the classification of sequence variants,^23^ this variant is reclassified as a likely pathogenic one (PS4, PM2, PP5, PP3, PP4).

**FIGURE 3:**
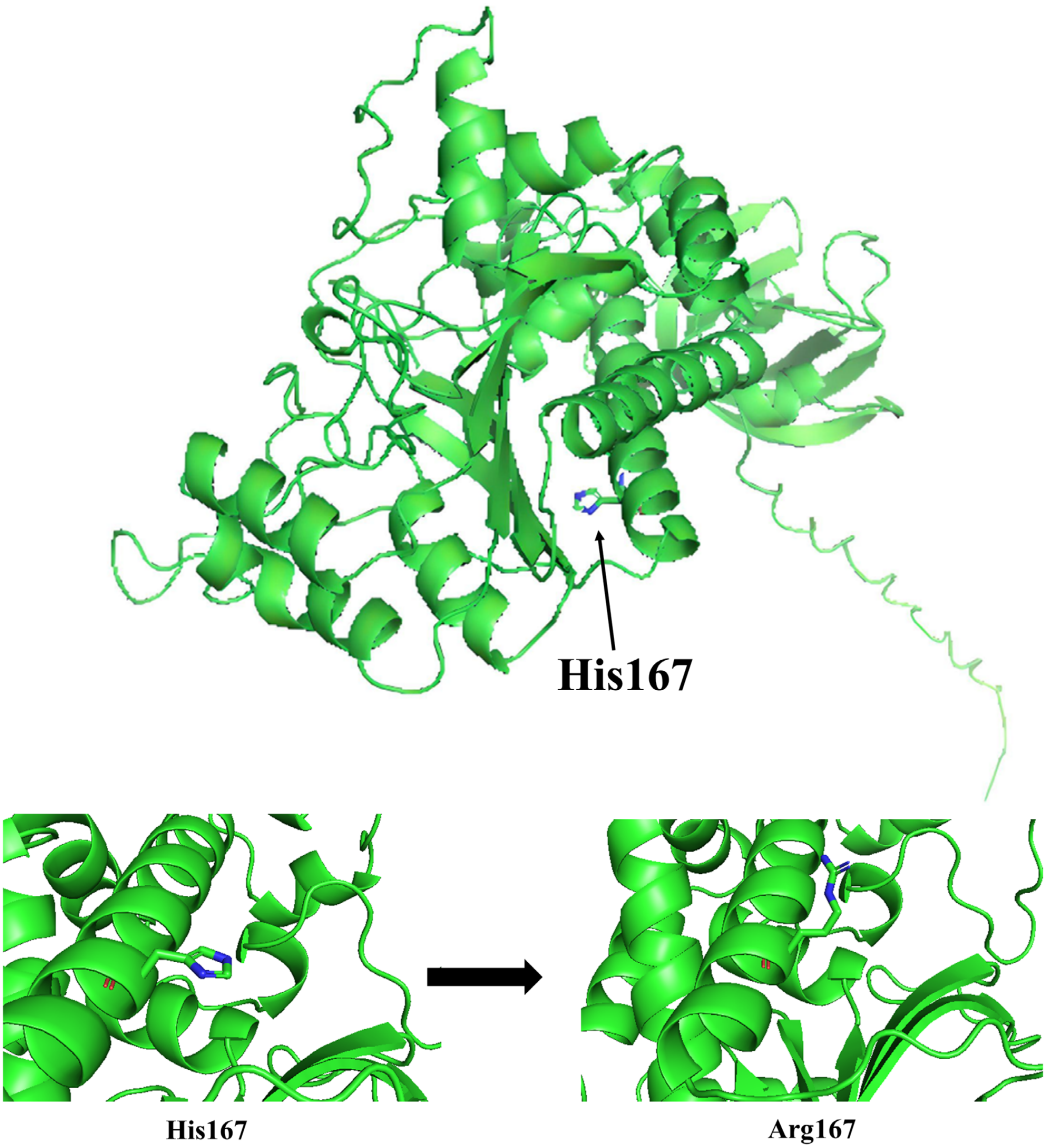
Protein 3D modeling of NARS2. (A) Schematic structure of NARS2. (B) Amino acid change and its effect on protein 3D structure.

Protein 3D modeling was performed to better understand the protein structure (Figure 3A). Amino acid change and its effect on protein 3D structure were assessed using PyMol (Figure 3B).^24^ Using I‐Mutant2.0, it was revealed that the p.(His167Arg) variant could decrease the stability of the protein, which results in protein dysfunction.^25^

## 3 DISCUSSION

In this study, we presented the phenotypes of two unrelated male patients who suffered from epileptic encephalopathy and severe neurodevelopmental delay in association with significant white matter signal changes, subdural hematoma, and cerebral parenchymal hemorrhage in one of these two patients with the same homozygous c.500A > G; p.(His167Arg) variant in *NARS2*. The c.500A > G variant had been previously reported in an Israeli boy by Mizuguchi et al.^9^ We also reviewed all reported patients with variants in *NARS2*. All data are summarized in a table to better understand the phenotypes associated with *NARS2* variants and their ACMG classification (Table 1).

All 3 patients with homozygote c.500A > G variant were born at term without any complications in pregnancy,^9^ although some other studies have reported pregnancy complications such as hyperemesis gravidarum,^10^ intrauterine growth retardation (IUGR)^6^ and breech position.^6^ Like the patient reported by Mizuguchi et al. in 2017 who manifested with generalized tonic–clonic and myoclonic seizures at 4 months of age, tonic spasm seizures in case 1 at 3.5 months old, and frequent seizures in case 2 at age of 3 months were observed. Similar to the patient reported by Mizuguchi et al., who suffered from intellectual disability and spastic quadriplegia, the first case had never achieved normal motor, speech, and cognitive milestones and experienced motor and cognition regression and loss of fix and follow ability and social smile. Besides, poor general condition, decreased level of consciousness, pyramidal, and extrapyramidal signs in this case were also observed. EEG in patient reported by Mizuguchi et al. showed a burst suppression pattern. The EEG of our first patient depicted generalized epileptic discharge, posterior polymorphic delta activity, and diffuse fast activity.^9^

From neurological examination standpoint, the patient reported by Mizuguchi et al. suffered from mild axial hypotonia at 4 months of age and presented with severe hypertonia and scissoring legs at 12 months. In this study, transient episodes of unilateral torticollis, dystonic postures, increased deep tendon reflexes, clonus, bilaterally upward plantar reflexes, moderate four limbs spasticity, especially in lower limbs, and notable hand fisting in the first patient, and truncal hypotoni and movement disorders in the second patient were observed. Therefore, movement disorders were documented more obviously in addition to pyramidal symptoms in our patients compared to the patient was reported by Mizuguchi et al. ABR showed bilateral sensorineural hearing loss in all three patients.^9^

In terms of imaging findings, brain MRI of the patient reported by Mizuguchi et al. showed cerebral atrophy with extended vacuolization of the periventricular white matter, basal ganglia, corpus callosum, and cerebellum at 9 months. In this study, brain MRI of the first patient at 16 months old revealed severe subcortical and deep cerebral and cerebellar white matter involvement, symmetric bilateral basal ganglia involvement, severe cerebral atrophy with large cystic necrotic areas in cerebral hemispheres, and severe thinning of the corpus callosum. Furthermore, subdural hematoma, cerebral parenchymal hemorrhage, and cerebellar cortical restriction in diffusion‐weighted sequences were novel imaging findings in this patient. The brain MRI of the second patient was normal at age of 7 months.^9^ Progressive cerebral atrophy and subdural space volume expansion could be an explanation for secondary subdural and intraparenchymal hemorrhage in the first patient. In a recently published paper describing imaging findings of a large cohort of 132 patients with different types of mitochondrial leukodystrophies, no cerebral hemorrhage was reported. However, the role of mitochondria in secondary brain injury and subarachnoid hemorrhage has been discussed in a systematic review that has been published by Zhang et al.^26^, ^27^, ^28^, ^29^

Electrolyte abnormalities and elevated blood lactate levels were detected in both patients. In the patient reported by Mizuguchi et al.^9^ only elevated serum lactate was reported. Our patients died at the age of 2 years and 1 year, respectively, due to respiratory failure, while in the Mizuguchi et al., the patient was alive at 4 years of age.^9^

Comparing the phenotype of patients, especially who carried same homozygous or compound heterozygous mutations could show the pleiotropic nature of NARS2‐related disorder, which may play an important role in disease diagnosis.

The most prevalent phenotype in the reported patients carrying homozygote c.641C > T, c.822G > C, c.1184 T > G, c.545 T > A, and compound heterozygote c.969 T > A and c.1142A > G, c.707 T > G and c.594 + 1G > A, c.1141A > G and c.1290G > C, c.1253G > A and c.1300C > T variants was different types of seizures including epileptic seizures, tonic–clonic seizures, myoclonic seizures.^6^, ^9^, ^10^, ^11^, ^13^, ^16^, ^17^, ^20^ Additionally, epilepsy phenotype was reported in patients carrying homozygote c.822G > C, c.631 T > A and compound heterozygote c.83_84del and c.1339A > G, c.707 T > G and c.594 + 1G > A variants.^7^, ^10^, ^23^, ^26^, ^27^ Developmental delay, intellectual impairment, psychomotor regression and cognitive impairment were also observed in most of patients carrying homozygote c.641C > T, c.822G > C, c.1184 T > G, c.545 T > A, c.951C > T and compound heterozygote c.707 T > G and c.594 + 1G > A, c.1141A > G and c.1290G > C variants.^6^, ^9^, ^10^, ^11^, ^12^, ^16^, ^19^, ^20^ Hearing impairment was another prevalent phenotype observed in patients carrying homozygote c.641C > T, c.637G > T, c.545 T > A, c.951C > T, c.658A > G and compound heterozygote c.969 T > A and c.1142A > G, c.707 T > G and c.594 + 1G > A, c.1141A > G and c.1290G > C variants.^6^, ^8^, ^9^, ^13^, ^15^, ^16^, ^19^, ^20^

Other clinical outcomes reported in assessed patients include muscle problems such as spastic quadriplegia, flaccid quadriplegia, myopathy, amyotrophy, scoliosis, hypotonia, severe hypoesthesia in lower limbs, severe hallux valgus in feet, upper limb tremor, brisk deep tendon reflexes, extensor plantar responses, clubbed fingers, brachymetatarsia, ataxic gait; visual impairment and optic atrophy; microcephaly, excessive fatigue and attention difficulties; kidney related disorders such as urinary retention, urinary incontinence and renal dysfunction; hemiparesis, pharyngeal hypotonia, laryngomalacia, feeding difficulties, dysarthria and dysphagia.^6^, ^9^, ^10^, ^11^, ^12^, ^13^, ^16^, ^19^ Some least prevalent clinical outcomes observed in some patients include frequent falls, premature menopause, multisystem mitochondrial disorder, intrauterine growth restriction, perinatal insult, progressive ataxia, hyperCKemia, gastrointestinal reflex, anemia, lactic acidosis, ketosis and hyperglycemia.^11^, ^12^, ^13^, ^14^, ^16^

Para clinical tests, such as laboratory tests, MRI, EEG and muscle biopsy results, were available for some of the patients. Increased blood lactate level was the most prevalent anomaly in laboratory results.^6^, ^9^, ^10^, ^13^, ^15^, ^19^

Although MRI results were not available for all patients, abnormal results were observed in some individuals. T2‐weighted hyperintensity in different parts of the brain, cerebral atrophy, restricted diffusion in the temporal lobe, left basal ganglia and cerebral cortex, diffuse atrophy, deep gray matter, subdural effusions, asymmetry of the hippocampus were the most brain disorders observed in available MRI results.^6^, ^7^, ^9^, ^10^, ^13^, ^14^, ^15^, ^19^

Bilateral synchronous spike and polyspike waves mainly in the posterior part of the brain, focal discharges, multifocal and spikes and sharp waves in addition to slow waves and irregular spikes in the central, parietal, and temporal regions were some of abnormal EEG results shown in some patients.^7^, ^16^, ^19^, ^20^

Muscle biopsy in some patients revealed slightly increased lipid/atrophic fibers, atrophic fibers, ragged red fibers, enlarged mitochondria, increased lipid profile, mitochondrial subsarcolemmal proliferation, diffuse presence of COX‐negative fibers and lipid droplets, decreased complexes II and subtle atrophy of the muscle fibers.^6^, ^7^, ^10^, ^11^, ^19^

Among all patients reviewed in this article, 8 patients died at the ages of 5.5, 6, 9, 14 and 15 months and 6, 16 years old.^6^, ^7^, ^10^, ^13^, ^15^, ^20^ Other patients were alive at the time of the review at the ages of 2 and 28 months and 1, 2, 8, 17, 22, 25, 26, 29 and 34 years old.^6^, ^9^, ^11^, ^12^, ^13^, ^16^, ^19^ Other patients were assessed in follow‐up studies.^8^, ^13^, ^17^, ^18^

As the previous studies reported the same variant, c.500A > G (p.His167Arg), the novel imaging phenotype, diffuse deep cerebral white matter signal changes, subdural hematoma, cerebral parenchymal hemorrhage, and cerebellar cortical restriction in diffusion weighted sequences, which was revealed in one of our patients, and well‐documented movement disorders, especially dystonia, could be considered as new spectrum of NARS2‐related disorders and it shows that more extensive investigations is needed to find out all aspects of this rare mitochondrial disorder. Although various disease phenotypes were reported related to *NARS2* variants, as the number of known cases is limited, genotype–phenotype correlation remains unidentified.

As congenital anomalies are increasingly the most significant causes of infant abnormality or death, accurate assessment of causative mutations could help affected families in using advanced molecular tools to prevent giving birth to affected individuals. In recent years, various molecular testing, such as array comparative genomic hybridization (CGH), single‐nucleotide polymorphism (SNP) array, quantitative real‐time PCR (qPCR), next‐generation sequencing (NGS) and non‐invasive prenatal testing (NIPT), have been developed to help families to have healthy children using preimplantation genetic diagnosis (PGD) and prenatal diagnosis (PND).^30^, ^31^, ^32^

## AUTHOR CONTRIBUTIONS

M.G and ART designed the project, M.Kh. and F.B. and E.A. did the data collection & analysis, M.Kh. and F.B. conducted the experiments, A.R.T., N.P., S.H., and M.Sh did the clinical evaluation, M.Kh., F.B., F.G. and M.F. wrote the paper. All authors read and approved the final manuscript.

## FUNDING INFORMATION

Research reported in this publication was supported and funded by the Tehran University of Medical Sciences (Grant number: 96‐02‐30‐35551) and the National Institute for Medical Research Development (electronic application No: 983886).

## CONFLICT OF INTEREST STATEMENT

The authors declare that they have no competing interests.

## Data Availability

The data that support the findings of this study are available on request from the corresponding author. The data are not publicly available due to privacy or ethical restrictions.
